# 2,2′-Bis(4-fluoro­anilino)-3,3′-(3,6-dioxa­octane-1,8-di­yl)diquinazolin-4(3*H*)-one

**DOI:** 10.1107/S1600536808040841

**Published:** 2008-12-10

**Authors:** Xiang Wang, Zuan Ma, Yu-Lu Chen

**Affiliations:** a Department of Chemistry, Kaili College, Guizhou 556000, People’s Republic of China; b Department of Medicinal Chemistry, Yunyang Medical College, Shiyan 442000, People’s Republic of China; c Department of Chemistry, Xianning Vocational and Technical College, Xianning 437100, People’s Republic of China

## Abstract

In the centrosymmetric title compound, C_34_H_30_F_2_N_6_O_4_, the dihedral angle between the quinazolinone and fluorobenzene ring planes are 71.00 (2) and 74.94 (2)° and an intra­molecular N—H⋯O interaction stabilizes the conformation. In the crystal, C—H⋯F and C—H⋯O links help to establish the packing.

## Related literature

For the biological activity of quinazolinones, see: Shiba *et al.* (1997 ▶); Ding *et al.*, 2004 ▶. For the crystal structures of other fused heterocyclic derivatives, see: Wang *et al.* (2006 ▶); Xu *et al.* (2006 ▶). 
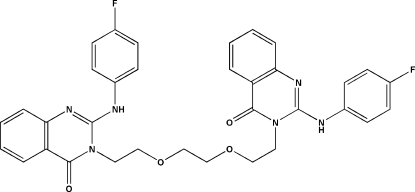


## Experimental

### 

#### Crystal data

C_34_H_30_F_2_N_6_O_4_
*M* *_r_* = 624.64
Monoclinic, 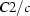

*a* = 13.923 (3) Å
*b* = 12.509 (3) Å
*c* = 18.726 (4) Å
β = 97.08 (3)°
*V* = 3236.6 (11) Å^3^
*Z* = 4
Mo *K*α radiation
μ = 0.10 mm^−1^
*T* = 295 (2) K
0.20 × 0.10 × 0.10 mm

#### Data collection

Bruker SMART 4K CCD area-detector diffractometer
Absorption correction: multi-scan (*SADABS*; Sheldrick, 2003 ▶) *T*_min_ = 0.982, *T*_max_ = 0.991
2834 measured reflections
2834 independent reflections
2263 reflections with *I* > 2σ(*I*)
*R*_int_ = 0.0123

#### Refinement

*R*[*F* ^2^ > 2σ(*F* ^2^)] = 0.048
*wR*(*F* ^2^) = 0.146
*S* = 1.06
2834 reflections
208 parameters
H-atom parameters constrained
Δρ_max_ = 0.30 e Å^−3^
Δρ_min_ = −0.16 e Å^−3^

### 

Data collection: *SMART* (Bruker, 2001 ▶); cell refinement: *SAINT-Plus* (Bruker, 2001 ▶); data reduction: *SAINT-Plus*; program(s) used to solve structure: *SHELXS97* (Sheldrick, 2008 ▶); program(s) used to refine structure: *SHELXL97* (Sheldrick, 2008 ▶); molecular graphics: *PLATON* (Spek, 2003 ▶); software used to prepare material for publication: *SHELXTL* (Sheldrick, 2008 ▶).

## Supplementary Material

Crystal structure: contains datablocks I, global. DOI: 10.1107/S1600536808040841/at2676sup1.cif

Structure factors: contains datablocks I. DOI: 10.1107/S1600536808040841/at2676Isup2.hkl

Additional supplementary materials:  crystallographic information; 3D view; checkCIF report

## Figures and Tables

**Table 1:** Hydrogen-bond geometry (Å, °)

*D*—H⋯*A*	*D*—H	H⋯*A*	*D*⋯*A*	*D*—H⋯*A*
N1—H1⋯O2	0.86	2.18	2.7954 (19)	128
C16—H16*A*⋯F1^i^	0.97	2.54	3.388 (2)	146
C16—H16*B*⋯O1^ii^	0.97	2.43	3.377 (2)	164

Symmetry codes: (i) 
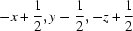
; (ii) 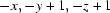.

**Fig. 1.**
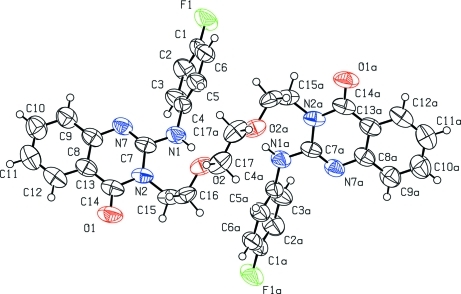
The molecular structure of the title compound, showing the atom-labeling scheme. Displacement ellipsoids are drawn at the 50% probability level.

**Fig. 2.**
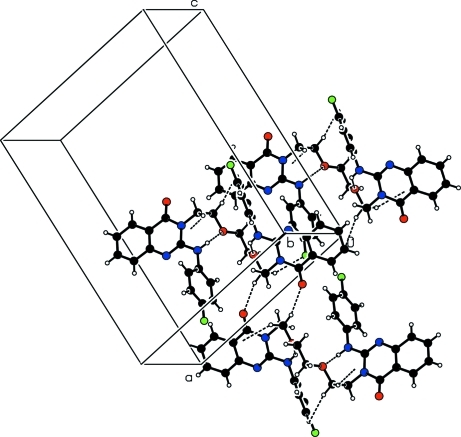
Packing diagram for title compound, showing the hydrogen bonds stacking interactions.

## supplementary crystallographic information

### Comment

Quinazolinones are important heterocycles exhibiting good biological and pharmaceutical activities. Some of these actities inclue antimicrobial, anti-inflammatory, antifungal, anticancer and AMPA receptor antagonistical properties (Shiba *et al.*, 1997 and Ding *et al.*, 2004). In connection with our ongoing heterocyclic synthesis and drug discovery project (Wang *et al.*, 2006; Xu *et al.*, 2006), we obtained the title compound by employing aza-Wittig reaction of beta-ethoxycarbonyl iminophosphorane with *p*-Flurophenyl isocyanate and subsequent 2-(2-(2-aminoethoxy)ethoxy)ethanamine under mild conditions. Herein, we present X-ray crystallographic analysis of the title compound, which may be used as a new precursor for obtaining bioactive molecules.

The selected bond lengths and angles are given in parameter see Table 1. In the molecule of the title compound (Fig. 1), the fused rings of quinazolinones are planar, and the phenyl (C1—C6) and (C1a—C6a) rings are twisted with respect to the two quinazolinone ring systems, making dihedral angles of 71.00 (2)° and 74.94 (2)°, respectively. The molecular conformation is stabilized by intermolecular N—H···O and O—H···N hydrogen bonds. In the crystal packing, intramolecular N—H···O and O—H···N hydrogen bonds and intermolecular C—H···F and C—H···O hydrogen bonds (Fig.2, Table 2) link the molecules, helping to stabilize the crystal structure.

### Experimental

To a solution of iminophosphorane (1.28 g, 3.0 mmol) in anhydrous THF (10 mL) was added p-Fluorophenyl isocyanate (0.41 g, 3.0 mmol) under nitrogen at room temperature. After standing for 10 h at 273-278K, the solvent was removed under reduced pressure and ethyl sther/petroleum ether (1:2, 10ml) was added to precipitate triphenylphosphine oxide. After filtration the solvent was removed to give the carbodiimide, which were used directly without further purification. To the solution of carbodiimide prepared above was added a solution of 2-(2-(2-aminoethoxy)ethoxy)ethanamine (3 mmol) in THF (10 mL). The mixture was stirred for 10 h at room temperature, concentrated under reduced pressure and the recrystallized from a mixed solvent of methanol and dichloromethane (1:2 v/v) at room temperature to give the title compound.

### Refinement

All H atoms were located in difference maps and treated as riding atoms with C—H = 0.93 Å, *U*_iso_=1.2*U*_eq_ (C) for C*sp*^2^, C—H = 0.97 Å, *U*_iso_ = 1.2*U*_eq_ (C) for CH_2_, N—H = 0.86 Å, *U*_iso_ = 1.2*U*_eq_ (N) for NH.

### Crystal data

**Table:**

C_34_H_30_F_2_N_6_O_4_	*F*(000) = 1304
*M**_r_* = 624.64	*D*_x_ = 1.282 Mg m^−^^3^
Monoclinic, *C*2/*c*	Melting point: 415 K
Hall symbol: -C 2yc	Mo *K*α radiation, λ = 0.71073 Å
*a* = 13.923 (3) Å	Cell parameters from 3566 reflections
*b* = 12.509 (3) Å	θ = 2.2–28.5°
*c* = 18.726 (4) Å	µ = 0.10 mm^−^^1^
β = 97.08 (3)°	*T* = 295 K
*V* = 3236.6 (11) Å^3^	Block, colourless
*Z* = 4	0.20 × 0.10 × 0.10 mm

### Data collection

**Table:**

Bruker SMART 4K CCD area-detector diffractometer	2834 independent reflections
Radiation source: fine-focus sealed tube	2263 reflections with *I* > 2σ(*I*)
graphite	*R*_int_ = 0.012
φ and ω scans	θ_max_ = 25.0°, θ_min_ = 2.2°
Absorption correction: multi-scan (*SADABS*; Sheldrick, 2003)	*h* = −16→16
*T*_min_ = 0.982, *T*_max_ = 0.991	*k* = 0→14
2834 measured reflections	*l* = 0→22

### Refinement

**Table:**

Refinement on *F*^2^	Secondary atom site location: difference Fourier map
Least-squares matrix: full	Hydrogen site location: inferred from neighbouring sites
*R*[*F*^2^ > 2σ(*F*^2^)] = 0.048	H-atom parameters constrained
*wR*(*F*^2^) = 0.146	*w* = 1/[σ^2^(*F*_o_^2^) + (0.0807*P*)^2^ + 0.8957*P*] where *P* = (*F*_o_^2^ + 2*F*_c_^2^)/3
*S* = 1.06	(Δ/σ)_max_ < 0.001
2834 reflections	Δρ_max_ = 0.30 e Å^−^^3^
208 parameters	Δρ_min_ = −0.15 e Å^−^^3^
0 restraints	Extinction correction: *SHELXL97* (Sheldrick, 2008)
Primary atom site location: structure-invariant direct methods	Extinction coefficient: 0.0028 (9)

### Special details

**Table:**

Geometry. All e.s.d.'s (except the e.s.d. in the dihedral angle between two l.s. planes) are estimated using the full covariance matrix. The cell e.s.d.'s are taken into account individually in the estimation of e.s.d.'s in distances, angles and torsion angles; correlations between e.s.d.'s in cell parameters are only used when they are defined by crystal symmetry. An approximate (isotropic) treatment of cell e.s.d.'s is used for estimating e.s.d.'s involving l.s. planes.
Refinement. Refinement of *F*^2^ against ALL reflections. The weighted *R*-factor *wR* and goodness of fit *S* are based on *F*^2^, conventional *R*-factors *R* are based on *F*, with *F* set to zero for negative *F*^2^. The threshold expression of *F*^2^ > σ(*F*^2^) is used only for calculating *R*-factors(gt) *etc*. and is not relevant to the choice of reflections for refinement. *R*-factors based on *F*^2^ are statistically about twice as large as those based on *F*, and *R*- factors based on ALL data will be even larger.

### Fractional atomic coordinates and isotropic or equivalent isotropic displacement parameters (Å^2^)

**Table:**

	*x*	*y*	*z*	*U*_iso_*/*U*_eq_	
C1	0.24654 (14)	0.79492 (18)	0.12370 (9)	0.0755 (6)	
C2	0.24527 (18)	0.85820 (18)	0.18204 (11)	0.0885 (7)	
H2	0.2639	0.9295	0.1808	0.106*	
C3	0.21577 (17)	0.81506 (16)	0.24356 (10)	0.0807 (6)	
H3	0.2140	0.8575	0.2841	0.097*	
C4	0.18901 (12)	0.70941 (14)	0.24493 (8)	0.0605 (4)	
C5	0.19174 (14)	0.64790 (16)	0.18485 (9)	0.0705 (5)	
H5	0.1740	0.5763	0.1858	0.085*	
C6	0.22039 (15)	0.69037 (18)	0.12292 (9)	0.0771 (6)	
H6	0.2218	0.6487	0.0819	0.093*	
C7	0.21556 (14)	0.65123 (13)	0.36984 (9)	0.0621 (4)	
C8	0.36454 (15)	0.65449 (13)	0.43630 (9)	0.0675 (5)	
C9	0.46390 (16)	0.67547 (17)	0.44028 (11)	0.0822 (6)	
H9	0.4893	0.7023	0.4003	0.099*	
C10	0.52371 (19)	0.65675 (19)	0.50253 (12)	0.0928 (7)	
H10	0.5895	0.6713	0.5044	0.111*	
C11	0.4878 (2)	0.61653 (19)	0.56277 (12)	0.0948 (7)	
H11	0.5292	0.6041	0.6048	0.114*	
C12	0.3909 (2)	0.59501 (16)	0.56025 (10)	0.0852 (6)	
H12	0.3666	0.5682	0.6007	0.102*	
C13	0.32810 (15)	0.61330 (13)	0.49687 (9)	0.0680 (5)	
C14	0.22605 (15)	0.58965 (14)	0.49317 (9)	0.0704 (5)	
C15	0.06541 (15)	0.60240 (16)	0.42379 (11)	0.0761 (6)	
H15A	0.0350	0.6577	0.3924	0.091*	
H15B	0.0468	0.6141	0.4714	0.091*	
C16	0.02768 (14)	0.49650 (16)	0.39706 (11)	0.0774 (6)	
H16A	0.0650	0.4397	0.4225	0.093*	
H16B	−0.0393	0.4887	0.4055	0.093*	
C17	−0.00666 (15)	0.39575 (15)	0.28882 (13)	0.0858 (6)	
H17A	−0.0751	0.3931	0.2939	0.103*	
H17B	0.0241	0.3331	0.3121	0.103*	
F1	0.27593 (11)	0.83760 (13)	0.06324 (6)	0.1146 (6)	
N1	0.15482 (12)	0.66559 (12)	0.30729 (7)	0.0698 (4)	
H1	0.0949	0.6478	0.3056	0.084*	
N2	0.17129 (11)	0.61347 (11)	0.42748 (7)	0.0639 (4)	
N7	0.30664 (12)	0.67140 (12)	0.37194 (7)	0.0676 (4)	
O1	0.18667 (12)	0.55312 (13)	0.54259 (7)	0.0937 (5)	
O2	0.03497 (9)	0.48969 (9)	0.32193 (7)	0.0748 (4)	

### Atomic displacement parameters (Å^2^)

**Table:**

	*U*^11^	*U*^22^	*U*^33^	*U*^12^	*U*^13^	*U*^23^
C1	0.0723 (11)	0.1035 (15)	0.0487 (9)	−0.0327 (10)	−0.0003 (8)	0.0192 (9)
C2	0.1115 (17)	0.0794 (13)	0.0736 (12)	−0.0417 (12)	0.0075 (11)	0.0167 (10)
C3	0.1106 (16)	0.0716 (12)	0.0617 (10)	−0.0257 (11)	0.0181 (10)	0.0020 (9)
C4	0.0631 (10)	0.0661 (10)	0.0522 (9)	−0.0140 (8)	0.0069 (7)	0.0097 (7)
C5	0.0824 (12)	0.0691 (11)	0.0603 (10)	−0.0180 (9)	0.0098 (9)	0.0049 (8)
C6	0.0859 (13)	0.0933 (14)	0.0526 (10)	−0.0194 (11)	0.0099 (9)	0.0005 (9)
C7	0.0863 (12)	0.0520 (9)	0.0514 (9)	−0.0073 (8)	0.0223 (8)	0.0021 (7)
C8	0.0941 (13)	0.0543 (10)	0.0550 (9)	−0.0094 (9)	0.0121 (9)	−0.0040 (7)
C9	0.0958 (15)	0.0788 (13)	0.0710 (12)	−0.0233 (11)	0.0065 (11)	−0.0028 (9)
C10	0.1032 (17)	0.0882 (15)	0.0835 (14)	−0.0154 (12)	−0.0025 (12)	−0.0092 (11)
C11	0.118 (2)	0.0860 (15)	0.0751 (14)	−0.0010 (14)	−0.0111 (13)	−0.0055 (11)
C12	0.128 (2)	0.0717 (12)	0.0567 (10)	0.0069 (12)	0.0138 (11)	0.0024 (9)
C13	0.0973 (14)	0.0537 (9)	0.0548 (9)	0.0057 (9)	0.0162 (9)	−0.0011 (7)
C14	0.1018 (15)	0.0591 (10)	0.0550 (9)	0.0125 (9)	0.0286 (9)	0.0075 (8)
C15	0.0861 (13)	0.0765 (12)	0.0737 (11)	0.0247 (10)	0.0416 (10)	0.0188 (9)
C16	0.0625 (11)	0.0819 (13)	0.0943 (13)	0.0092 (9)	0.0359 (10)	0.0323 (10)
C17	0.0731 (12)	0.0562 (10)	0.1317 (18)	−0.0043 (9)	0.0273 (12)	0.0129 (10)
F1	0.1282 (11)	0.1552 (13)	0.0596 (7)	−0.0636 (9)	0.0091 (7)	0.0307 (7)
N1	0.0761 (10)	0.0793 (10)	0.0556 (8)	−0.0195 (8)	0.0152 (7)	0.0120 (7)
N2	0.0824 (10)	0.0582 (8)	0.0561 (8)	0.0061 (7)	0.0290 (7)	0.0089 (6)
N7	0.0820 (11)	0.0696 (9)	0.0527 (8)	−0.0186 (8)	0.0142 (7)	0.0019 (6)
O1	0.1137 (11)	0.1071 (11)	0.0679 (8)	0.0187 (9)	0.0419 (8)	0.0301 (8)
O2	0.0723 (8)	0.0614 (7)	0.0970 (10)	−0.0068 (6)	0.0353 (7)	0.0114 (6)

### Geometric parameters (Å, °)

**Table:**

C1—C2	1.351 (3)	C10—H10	0.9300
C1—C6	1.357 (3)	C11—C12	1.371 (3)
C1—F1	1.3597 (19)	C11—H11	0.9300
C2—C3	1.380 (3)	C12—C13	1.403 (3)
C2—H2	0.9300	C12—H12	0.9300
C3—C4	1.374 (3)	C13—C14	1.445 (3)
C3—H3	0.9300	C14—O1	1.221 (2)
C4—C5	1.368 (2)	C14—N2	1.397 (2)
C4—N1	1.4239 (19)	C15—N2	1.474 (2)
C5—C6	1.379 (2)	C15—C16	1.489 (3)
C5—H5	0.9300	C15—H15A	0.9700
C6—H6	0.9300	C15—H15B	0.9700
C7—N7	1.289 (2)	C16—O2	1.426 (2)
C7—N1	1.369 (2)	C16—H16A	0.9700
C7—N2	1.390 (2)	C16—H16B	0.9700
C8—N7	1.381 (2)	C17—O2	1.419 (2)
C8—C13	1.397 (2)	C17—C17^i^	1.488 (5)
C8—C9	1.401 (3)	C17—H17A	0.9700
C9—C10	1.367 (3)	C17—H17B	0.9700
C9—H9	0.9300	N1—H1	0.8600
C10—C11	1.384 (3)		
			
C2—C1—C6	122.90 (16)	C11—C12—H12	119.8
C2—C1—F1	118.59 (19)	C13—C12—H12	119.8
C6—C1—F1	118.50 (19)	C8—C13—C12	119.8 (2)
C1—C2—C3	118.74 (19)	C8—C13—C14	119.35 (17)
C1—C2—H2	120.6	C12—C13—C14	120.89 (18)
C3—C2—H2	120.6	O1—C14—N2	119.99 (19)
C4—C3—C2	120.08 (19)	O1—C14—C13	124.83 (18)
C4—C3—H3	120.0	N2—C14—C13	115.17 (15)
C2—C3—H3	120.0	N2—C15—C16	114.13 (15)
C5—C4—C3	119.38 (16)	N2—C15—H15A	108.7
C5—C4—N1	120.27 (15)	C16—C15—H15A	108.7
C3—C4—N1	120.29 (16)	N2—C15—H15B	108.7
C4—C5—C6	121.04 (18)	C16—C15—H15B	108.7
C4—C5—H5	119.5	H15A—C15—H15B	107.6
C6—C5—H5	119.5	O2—C16—C15	108.66 (14)
C1—C6—C5	117.86 (18)	O2—C16—H16A	110.0
C1—C6—H6	121.1	C15—C16—H16A	110.0
C5—C6—H6	121.1	O2—C16—H16B	110.0
N7—C7—N1	120.14 (14)	C15—C16—H16B	110.0
N7—C7—N2	124.86 (16)	H16A—C16—H16B	108.3
N1—C7—N2	115.00 (16)	O2—C17—C17^i^	109.48 (13)
N7—C8—C13	122.25 (18)	O2—C17—H17A	109.8
N7—C8—C9	118.96 (17)	C17^i^—C17—H17A	109.8
C13—C8—C9	118.75 (18)	O2—C17—H17B	109.8
C10—C9—C8	120.5 (2)	C17^i^—C17—H17B	109.8
C10—C9—H9	119.8	H17A—C17—H17B	108.2
C8—C9—H9	119.8	C7—N1—C4	121.22 (15)
C9—C10—C11	120.9 (2)	C7—N1—H1	119.4
C9—C10—H10	119.5	C4—N1—H1	119.4
C11—C10—H10	119.5	C7—N2—C14	120.67 (16)
C12—C11—C10	119.8 (2)	C7—N2—C15	122.16 (15)
C12—C11—H11	120.1	C14—N2—C15	117.09 (14)
C10—C11—H11	120.1	C7—N7—C8	117.59 (15)
C11—C12—C13	120.3 (2)	C17—O2—C16	113.94 (14)
			
C6—C1—C2—C3	−0.3 (4)	C8—C13—C14—N2	−1.5 (2)
F1—C1—C2—C3	−179.8 (2)	C12—C13—C14—N2	178.81 (16)
C1—C2—C3—C4	0.4 (4)	N2—C15—C16—O2	−71.71 (19)
C2—C3—C4—C5	−0.1 (3)	N7—C7—N1—C4	−4.2 (3)
C2—C3—C4—N1	−177.47 (19)	N2—C7—N1—C4	176.50 (15)
C3—C4—C5—C6	−0.4 (3)	C5—C4—N1—C7	113.3 (2)
N1—C4—C5—C6	176.98 (17)	C3—C4—N1—C7	−69.4 (2)
C2—C1—C6—C5	−0.2 (3)	N7—C7—N2—C14	−2.9 (3)
F1—C1—C6—C5	179.32 (17)	N1—C7—N2—C14	176.34 (15)
C4—C5—C6—C1	0.5 (3)	N7—C7—N2—C15	173.61 (17)
N7—C8—C9—C10	−178.14 (18)	N1—C7—N2—C15	−7.1 (2)
C13—C8—C9—C10	−0.5 (3)	O1—C14—N2—C7	−177.52 (16)
C8—C9—C10—C11	0.2 (3)	C13—C14—N2—C7	3.6 (2)
C9—C10—C11—C12	0.0 (4)	O1—C14—N2—C15	5.8 (3)
C10—C11—C12—C13	0.2 (3)	C13—C14—N2—C15	−173.17 (15)
N7—C8—C13—C12	178.24 (16)	C16—C15—N2—C7	89.7 (2)
C9—C8—C13—C12	0.7 (3)	C16—C15—N2—C14	−93.65 (19)
N7—C8—C13—C14	−1.5 (3)	N1—C7—N7—C8	−179.41 (15)
C9—C8—C13—C14	−179.00 (17)	N2—C7—N7—C8	−0.2 (3)
C11—C12—C13—C8	−0.5 (3)	C13—C8—N7—C7	2.4 (3)
C11—C12—C13—C14	179.17 (19)	C9—C8—N7—C7	179.87 (17)
C8—C13—C14—O1	179.66 (17)	C17^i^—C17—O2—C16	−178.87 (17)
C12—C13—C14—O1	−0.1 (3)	C15—C16—O2—C17	−174.79 (15)

Symmetry codes: (i) −*x*, *y*, −*z*+1/2.

### Hydrogen-bond geometry (Å, °)

**Table:**

*D*—H···*A*	*D*—H	H···*A*	*D*···*A*	*D*—H···*A*
N1—H1···O2	0.86	2.18	2.7954 (19)	128
C16—H16A···F1^ii^	0.97	2.54	3.388 (2)	146
C16—H16B···O1^iii^	0.97	2.43	3.377 (2)	164

Symmetry codes: (ii) −*x*+1/2, *y*−1/2, −*z*+1/2; (iii) −*x*, −*y*+1, −*z*+1.
